# Safety and efficacy of topical testosterone in breast cancer patients receiving ovarian suppression and aromatase inhibitor therapy

**DOI:** 10.1186/s13058-024-01886-7

**Published:** 2024-09-16

**Authors:** Patrícia Taranto, Diogo de Brito Sales, Fernando Cotait Maluf, Rafael Aliosha Kaliks Guendelmann, Luciano de Melo Pompei, Alessandro Leal, Antonio Carlos Buzaid, Gustavo Schvartsman

**Affiliations:** 1 Centro de Oncologia e Hematologia Einstein Dayan-Daycoval, Hospital Israelita Albert Einstein, Sao Paulo, Brazil; 2 Hospital Israelita Albert Einstein, Goiânia, Brazil; 3 Faculdade de Medicina do ABC, São Paulo, Brazil; 4 NYU Langone Health, NY, USA; 5 Centro Oncológico Antonio Ermírio de Morais, Beneficência Portuguesa, São Paulo, Brazil

**Keywords:** Breast Cancer, Menopause, Libido, Testosterone, Aromatase inhibitors

## Abstract

**Background:**

Premenopausal, high-risk, hormone receptor-positive breast cancer patients are often treated with ovarian suppression in combination with aromatase inhibitors (AI). This combination has important adverse effects, particularly in sexual function, such as vaginal dryness and loss of libido. There is no effective therapy for reduced sexual function in this setting. Our study aimed to determine the efficacy and safety, particularly regarding sexual function, of a low-dose, topical testosterone gel administration.

**Methods:**

This is a pilot, single-center study, designed to evaluate the efficacy of topical testosterone gel (3 mg/day) in improving sexual function in 29 premenopausal patients on ovarian suppression in combination with an AI. The primary safety endpoint was to assess serum estradiol elevation. The primary efficacy endpoint was sexual function improvement, assessed by the Female Sexual Function Index questionnaire.

**Results:**

We report the results on 29 patients. Twenty-two patients (75%) completed the 3-month treatment, and seven discontinued treatment before completion, mostly due to logistical difficulties related to the COVID-19 pandemic. All patients maintained the value of baseline mass spectrometry assay for estradiol of less than 2.7 pg/mL during the undertaken measurements. We observed a significant improvement in *Female Sexual Function Index* measures over the visits, with an increase from a mean of 11.7 at baseline to 19.1 in the third month (*p* < 0.001), with the greatest improvement observed between the second and third months.

**Conclusions:**

Our findings suggest that topical testosterone seems to be safe and may be effective in improving sexual function in patients on ovarian suppression and AI.

**Trial registration:**

The project was submitted and approved through the hospital’s SGPP platform in 11/26/2019 (Project No. SGPP 393819) and CAAE (Research Ethics Committee) (CAAE No 25609719.5.0000.007).

## Introduction

The treatment of early-stage, hormone receptor-positive breast cancer includes surgery, chemotherapy and/or radiotherapy, followed by hormone therapy, particularly with aromatase inhibitors (AI) and tamoxifen for at least 5 years [1]. In premenopausal patients at high risk of recurrence, the standard treatment consists of ovarian suppression to block hormonal production through the gonadal pathway, combined with tamoxifen or AI [2].

Recently, a meta-analysis confirmed the benefit of ovarian suppression in the treatment of premenopausal patients, especially those who remained premenopausal after chemotherapy or who did not undergo chemotherapy, with improved survival and lower recurrence rates in patients who were treated with non-tamoxifen-based hormonal therapy [3]. However, the use of AI increases the frequency of hormonal side effects, leading to worsening of vaginal dryness, loss of libido, and other symptoms that significantly affect quality of life [4]. These symptoms are often more pronounced in premenopausal women treated with ovarian suppression [5] due to the abrupt chemical menopause.

The treatment of vaginal atrophy and loss of libido remains a significant challenge. Intravaginal estriol or testosterone may mitigate local adverse effects with no systemic increase in estradiol levels, but consequently lack systemic effect [6–8]. Systemic estrogen/progesterone replacement improves symptoms attributed to hormone deprivation, but may increase the risk of breast cancer development and recurrence [9–15].

As for testosterone, the data is scarce regarding systemic replacement to improve symptoms attributed to hormone therapy [6, 7]. Testosterone is converted to estradiol by the activity of the aromatase enzyme, thus potentially increasing the risk of breast cancer recurrence. The use of AIs, however, may prevent such conversion and provide safety for this strategy. Prompted by this unmet need, we conducted a pilot study designed to evaluate the efficacy and safety of topical testosterone gel (3 mg/day) in the skin, with systemic absorption, in improving sexual function for premenopausal women with breast cancer receiving ovarian suppression and AIs, without increasing estradiol levels.

## Methods

This is a prospective, single-arm intervention pilot study involving 29 premenopausal patients with hormone-positive breast cancer undergoing ovarian suppression and aromatase inhibitor therapy. The study was conducted at the Center for Oncology and Hematology Família Dayan Daycoval, at Hospital Israelita Albert Einstein. The selection of only premenopausal women was based on the greater symptomatology experienced by patients during hormonal blockade compared to postmenopausal women. The project received a grant from AMIGOH project for its implementation.

### Objectives

The primary objective was to evaluate the effect of low-dose topical testosterone supplementation (3 mg/mL) on serum estradiol levels measured by mass spectrometry in premenopausal patients with breast cancer undergoing ovarian suppression and aromatase inhibitor therapy.

The secondary objective was to assess the efficacy of topical testosterone in improving sexual symptoms such as libido and vaginal dryness.

### Inclusion criteria

We included female, premenopausal patients with hormone-positive breast cancer receiving adjuvant or metastatic treatment with ovarian suppression and aromatase inhibitors for at least three months, experiencing symptoms related to hormone therapy. Baseline E2 (estradiol) levels had to be < 2.7 pg/mL.

### Exclusion criteria

We excluded patients with any contraindication or hypersensitivity to topical testosterone use, known pre-existing psychiatric disorder, history of alcohol or illicit drug abuse, and use of antidepressants or any other medication that may influence sexual dysfunction that was initiated within three months before enrollment in the study protocol. Use of other medications containing any type of hormone or intravaginal laser therapy within the last 15 days before the patient’s initiation in the protocol was also prohibited.

### Study design

**Fig. 1:**
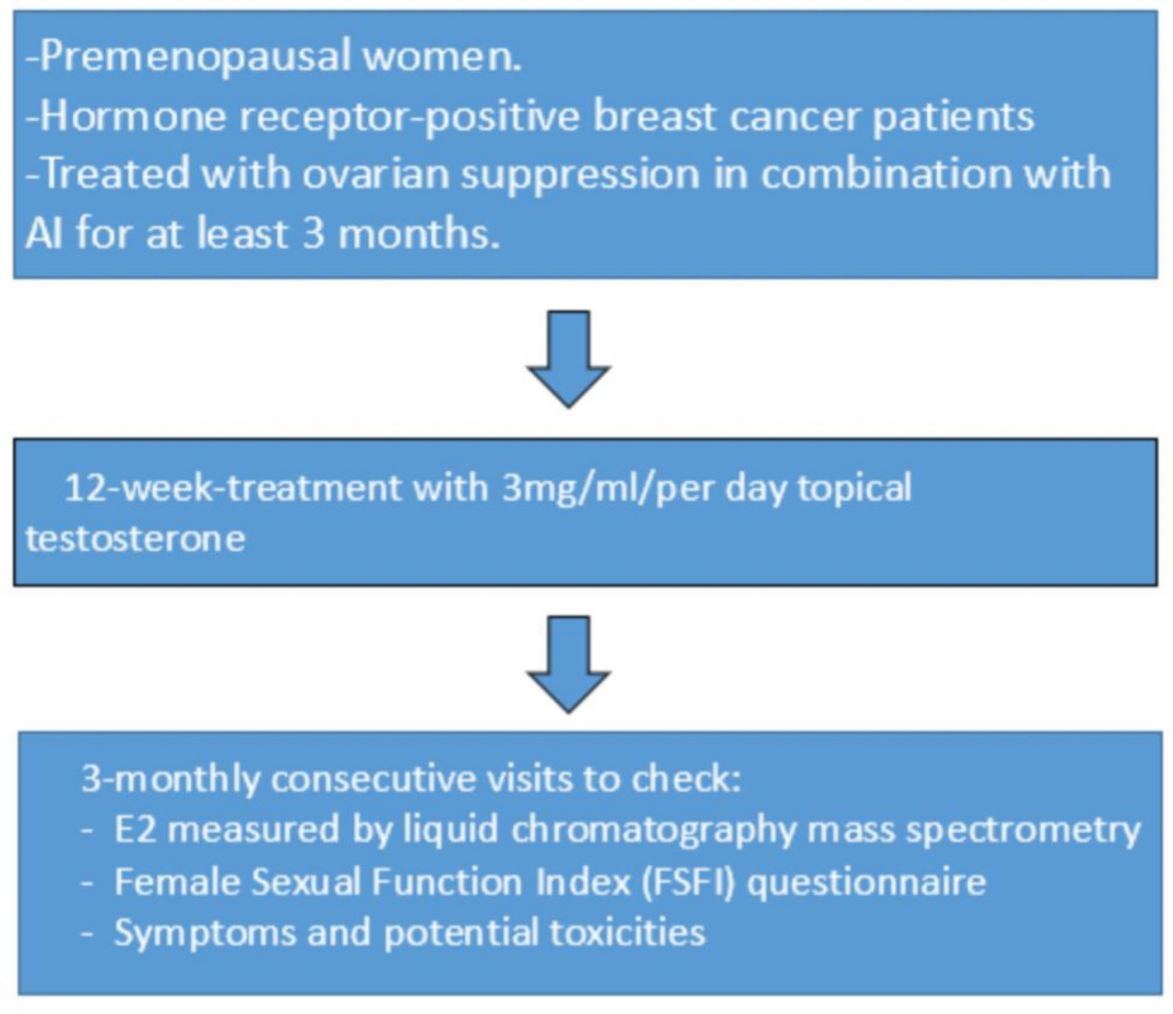
Study design with the inclusion criteria, treatment and 3-month follow-up during the evaluating the effect of topical testosterone use on the the elevation of estradiol levels

This is a pilot, single-arm, prospective, interventionist study aimed at evaluating the effect of topical testosterone use on the elevation of estradiol levels (Fig. 1). The treatment consists of daily applications of topical testosterone gel at a dose of 1 pump (equivalent to 1mL containing 3 mg of testosterone) on the skin of the inner thigh for 12 weeks. The safety of the treatment is assessed through monthly measurements of E2 during the treatment period. E2 values < 2.72 pg/mL are considered appropriate and safe for the continuation of treatment. Additionally, possible changes in sexual symptoms, such as libido, were evaluated using the Female Sexual Function Index (FSFI) questionnaire. This questionnaire, validated in Portuguese, consists of 19 questions with responses ranging from 0 to 5, indicating increasing levels of function in the areas assessed. It aims to assess the woman’s sexual function in the past four weeks, particularly in relation to female sexual response across six main domains: sexual desire, sexual arousal, vaginal lubrication, orgasm, sexual satisfaction, and pain [16]. The questionnaire generates a final score ranging from 2 to 36, with higher scores indicating better perception of the patient’s sexual function across the mentioned domains (Table 1). At each clinic visit, the patients completed the FSFI questionnaire and underwent serum estradiol measurements using mass spectrometry. The scheduled visits for each patient were distributed as follows: the first visit (visit 1) for inclusion interview, protocol explanation, and signing of the informed consent form (ICF), followed by visits with a monthly interval for baseline FSFI questionnaire administration, estradiol evaluation by mass spectrometry, and reapplication of the questionnaire (visits 2–4). During these visits, patients were also questioned about the progression of hormone therapy symptoms and possible side effects of the treatment. After the recruitment of patients, the questionnaires were analyzed along with the results of the E2 tests. Patients with E2 measurements > 2.72 pg/mL in at least 2 assessments during testosterone replacement treatment would be excluded from the study. If this increase occurred in more than 5% of the total recruited patients, the study would be terminated. Participants signed the ICF at the beginning of the recruitment, and the study was evaluated and approved by the Research Ethics Committee of the Hospital Israelita Albert Einstein.

**Table 1:** Calculation of sexual function scores

Score	Formula	Mínimum value	Maximum value
Sexual desire	0,6*sum (f01, f02)	1,2	6
Arousal	0,3*sum (f03, f04, f05, f06)	0	6
Lubrification	0,3*sum (f07, f08, f09, f10)	0	6
Orgasm	0,4*sum (f11, f12, f13)	0	6
Satisfaction	0,4*sum (f14, f15, f16)	0,8	6
Pain	0,4*sum (f17, f18, f19)	0	6
**Total score**	**Sum of scores across all domains**	**2**,**0**	**36**

In the article a total score is not mentioned, however we kept this because it was calculated in the database by the researcher

### Statistical methods planning

Categorical variables were described using absolute frequencies and percentages, while numerical variables were presented as means and standard deviations, or medians and quartiles, along with minimum and maximum values [17]. The distributions of numerical variables were assessed using histogram and boxplot graphs, as well as the Shapiro-Wilk [18] normality test. To investigate variations in the FSFI instrument score between visits, a generalized estimating equation model with Tweedie distribution and autoregressive correlation structure of order 1 was adjusted, and the results were presented as estimated means, 95% confidence intervals, and *p*-values for comparisons with the value at visit 1. The analyses were performed using the SPSS software, considering a significance level of 5%. The data were collected as described in the article by Pacagnella et al., with questions 17, 18, and 19 reversed [19]. The scoring of the FSFI scores was also calculated according to the recommendations of the article, as follows:

### Sample size estimation

The study was conducted with a convenience sample of 29 women. No formal sample size calculation was performed as this was a pilot study.

## Results

**Fig. 2:**
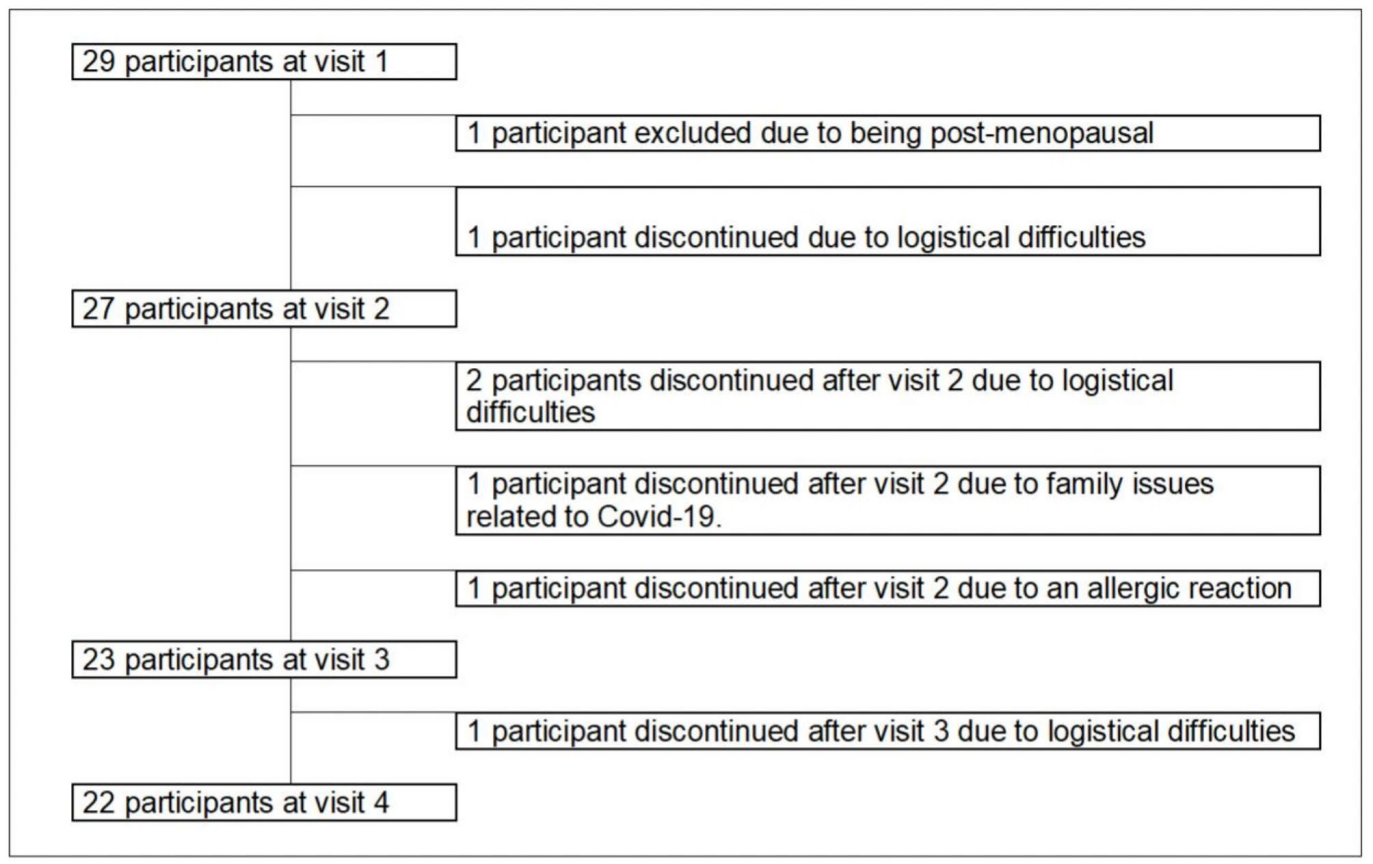
Consort diagram: twenty-nine patients were included. After 3 months, 22 completed the full course of treatment and 7 discontinued therapy before study completion

A total of twenty-nine patients were included and started the experimental treatment, of whom 22 completed the 3-month treatment. Two patients discontinued treatment prior to the first blood collection at Visit 2 (one was excluded upon discovering she was postmenopausal and the other withdrew consent due to logistical difficulties during the COVID-19 pandemic); four additional patients discontinued treatment between Visits 2 and 3 (three because of logistical difficulties and one due to a mild skin reaction, which was resolved after discontinuation). Between Visits 3 and 4, another patient withdrew consent, due to the pandemic (Fig. 2).

The age of the participants ranged from 30.7 to 51.4 years, with a mean age of 40.7 years. The most frequent T stage was T2 (46.2%), and the most common nodal stage was N1a (30.8%). Three patients had metastatic disease (Table 2). Regarding treatment characteristics, 22 (84.6%) received adjuvant chemotherapy, and 21 (80.8%) received adjuvant radiotherapy. The most common hormone therapy regimen was goserelin + exemestane, in 14 cases (53.8%).

**Table 2:** Characteristics of participants and treatment

Demographic Data	
Age (years)	
Mean (standard deviation)	40,7 (6,2)
Minimum – Maximum	30,7–51,4
T-stage, n (%)	
1ª	2 (7,7)
1b	3 (11,5)
1c	3 (11,5)
2	12 (46,2)
3	5 (19,2)
X	1 (3,8)
Number of affected lymph nodes, n (%)	
0	5 (19,2)
0(i+)	1 (3,8)
1mi	3 (11,5)
1a	8 (30,8)
1b	4 (15,4)
2a	3 (11,5)
2b	1 (3,8)
X	1 (3,8)
Presence of metastasis, n (%)	
No	23 (88,5)
Yes	3 (11,5)
Surgery, n (%)	
Yes	25 (96,2)
No	1 (3,8)
Adjuvant chemotherapy,, n (%)	
Yes	22 (84,6)
No	4 (15,4)
Adjuvant chemotherapy regimen, n (%)	
ACdd + T	10 (45,5)
TC x 6	5 (22,7)
AC + T	1 (4,5)
Other	6 (27,3)
Total	22 (100,0)
If other regimen, which one? n (%)	
ddEC-T	1 (16,7)
EC-T	1 (16,7)
Taxol–> FEC	1 (16,7)
TCH	1 (16,7)
TCH x6	1 (16,7)
TCHP adjuvant	1 (16,7)
Total	6 (100,0)
Adjuvant Hormone Therapy Regimen, n (%)	
Goserelin + Exemestane	14 (53,8)
Goserelin + Letrozole	9 (34,6)
Goserelin + Tamoxifen	1 (3,8)
Goserelin + Exemestane	1 (3,8)
Goserelin + Palbociclib + Anastrozole	1 (3,8)
Letrozol	1 (3,8)
Received adjuvant radiotherapy, n (%)	
Yes	21 (80,8)
No	5 (19,2)

*n* = 29

### Safety

Of the 27 evaluated patients with at least one blood collection after treatment initiation, all maintained E2 values < 2.7 pg/mL throughout their measurements. Toxicity was not formally assessed as treatment duration was of short term and we only observed transient, low-grade reactions in the skin.

### Efficacy

**Fig. 3:**
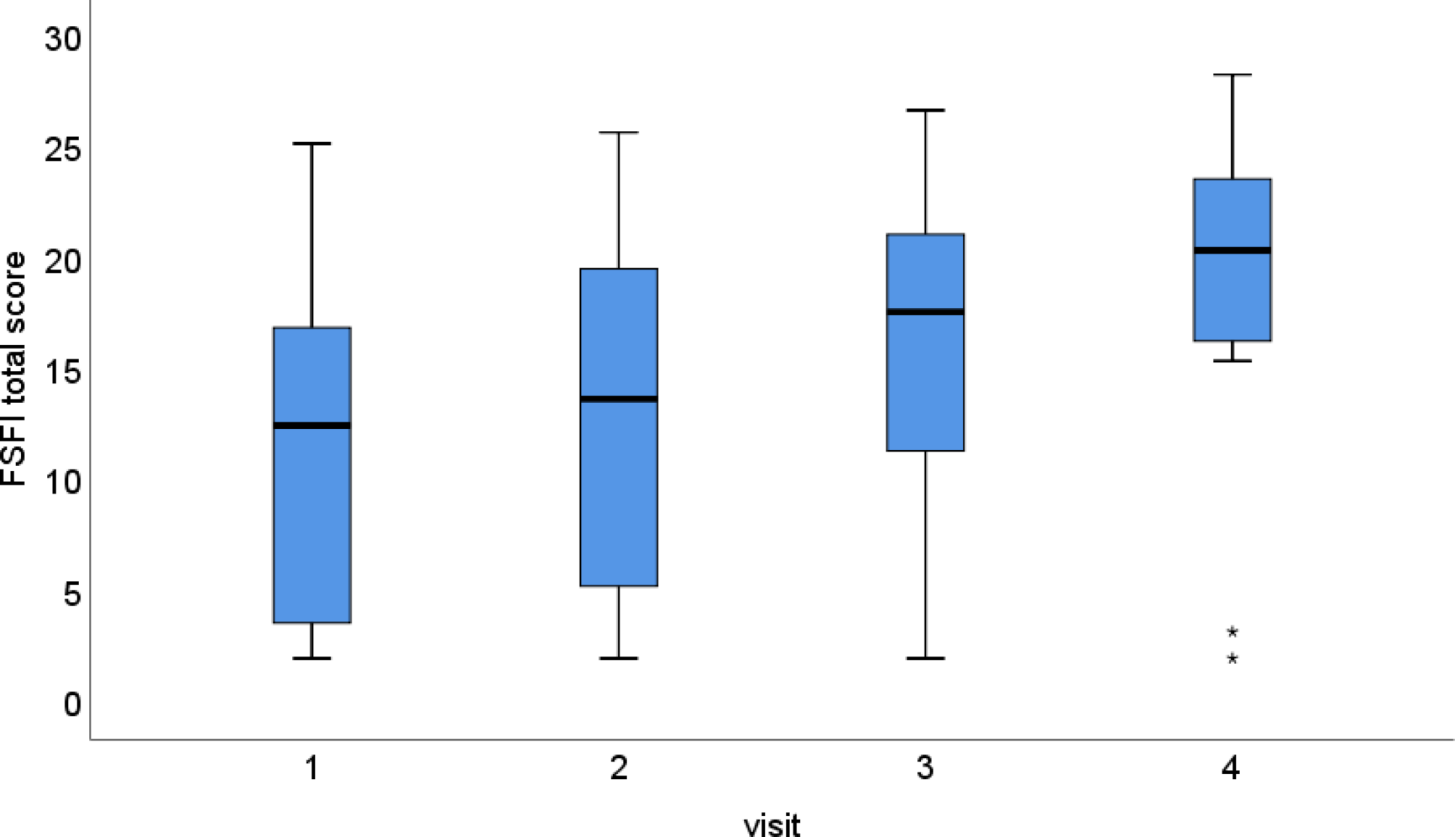
Significant increase in FSFI scores over time. At Visit 1, the score was 11.7. At Visit 4, the score increased to 19.1 (*P* < 0.001)

We observed a significant increase in FSFI scores over time, with significant differences starting from Visit 3. The estimated mean score at Visit 1 was 11.7, which increased to 16.3 at Visit 3 and 19.1 at Visit 4 (*P* < 0.001; Fig. 3).

**Fig. 4:**
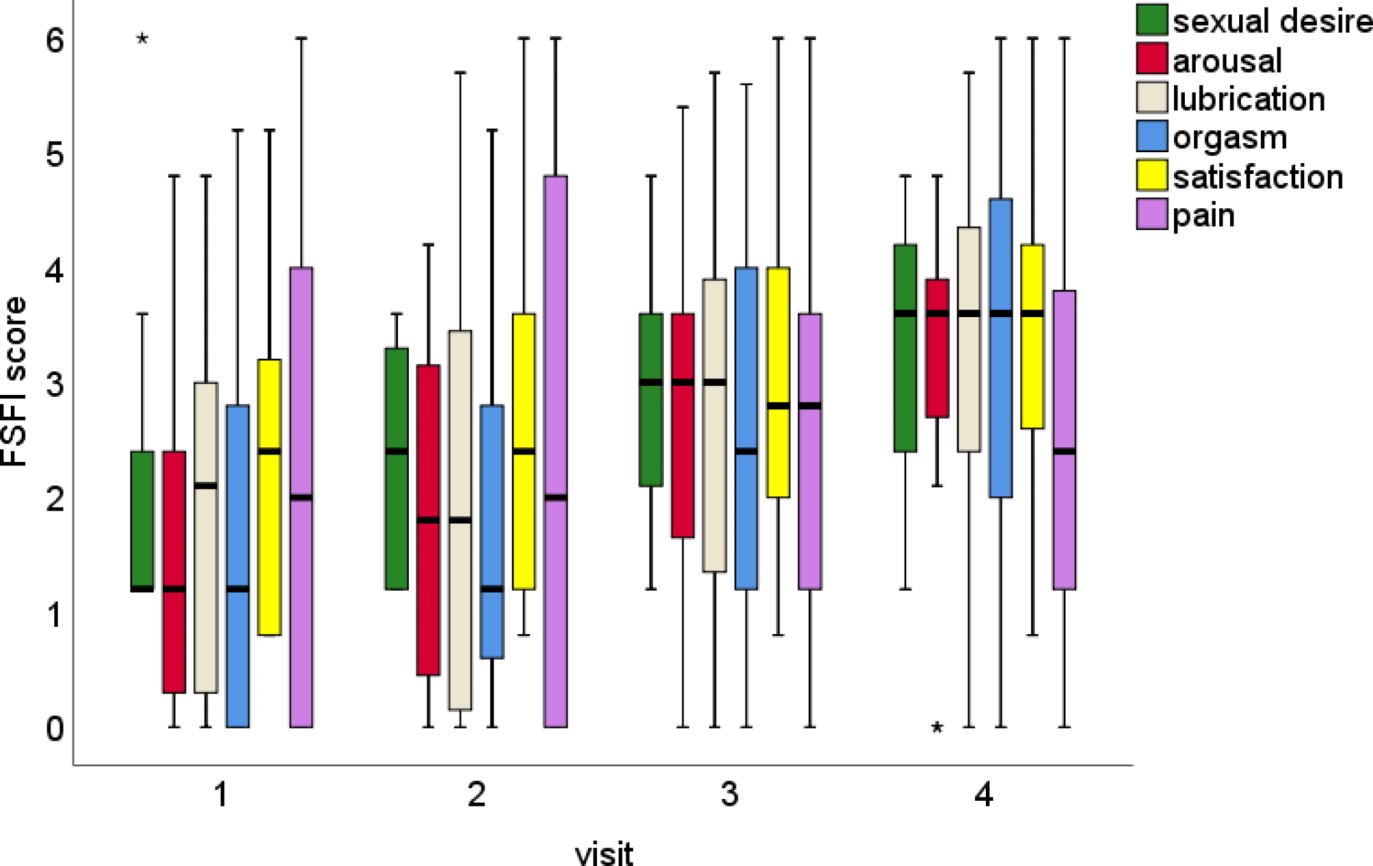
Significant increase in nearly all FSFI domains score over the visits

The adjusted models to assess the evolution of sexual function scores show that for desire, arousal, lubrication, orgasm, satisfaction, and total FSFI score, there is a significant increase from Visit 1 starting from Visit 3. Regarding pain, we did not observe significant changes compared to Visit 1 (Fig. 4; Table 3).

**Table 3:** Evolution of sexual function measures across visits

	Visit 1	Visit 2	Visit 3	Visit 4
Sexual desire				
Mean *	1,9 (1,5; 2,3)	2,3 (2,0; 2,7)	2,8 (2,4; 3,2)	3,3 (2,8; 3,7)
*p*-value**	Reference	0,063	0,001	< 0,001
Arousal				
Mean *	1,6 (1,1; 2,1)	1,9 (1,4; 2,4)	2,6 (2,1; 3,1)	3,1 (2,6; 3,6)
*p*-value**	Reference	0,101	< 0,001	< 0,001
Lubrication				
Mean *	1,9 (1,3; 2,4)	2,1 (1,4; 2,8)	2,8 (2,1; 3,5)	3,2 (2,6; 3,8)
*p*-value**	Reference	0,487	0,018	< 0,001
Orgasm				
Mean*	1,6 (1,0; 2,2)	1,9 (1,3; 2,5)	2,5 (1,8; 3,1)	3,2 (2,4; 4,0)
*p*-value**	Reference	0,172	0,001	< 0,001
Satisfaction				
Mean *	2,4 (1,9; 2,9)	2,6 (2,0; 3,2)	3,2 (2,6; 3,7)	3,5 (2,8; 4,1)
*p*-value**	Reference	0,566	0,003	0,009
Pain				
Mean *	2,3 (1,5; 3,0)	2,4 (1,6; 3,2)	2,5 (1,8; 3,2)	2,9 (2,1; 3,6)
*p*-value**	Reference	0,736	0,588	0,244
FSFI total score				
Mean (95% CI)*	11,7 (9,1; 14,3)	13,1 (10,4; 15,9)	16,3 (13,7; 18,9)	19,1 (16,3; 21,9)
*p*-value**	Reference	0,190	< 0,001	< 0,001

*Means and confidence intervals estimated by mixed models (95% CI)

**: *p*-value for comparisons relative to visit 1

FSFI: Female Sexual Function Index

## Discussion

Patients undergoing treatment for breast cancer with ovarian suppression and aromatase inhibitors experience significant hormonal side effects due to hormonal deprivation, such as vaginal dryness and loss of libido. Our study demonstrated the feasibility of delivering low doses of systemic testosterone to improve sexual function without increasing estradiol levels.

The treatment of vaginal atrophy and loss of libido remains a significant challenge. The safety of intravaginal testosterone use has been evaluated in women with breast cancer. Overall, the studies demonstrate no significant effect on serum estradiol levels, despite some degree of systemic testosterone absorption [6, 7]. The improvement associated with its application, however, was mostly limited to local effects, such as vaginal atrophy and sexual function, with no significant impact on systemic effects of ovarian suppression, such as hot flashes, metabolic changes, sleep disturbances, and irritability. Similarly, the use of low-dose intravaginal estriol (0.005%) has also been evaluated in women with breast cancer undergoing adjuvant treatment with AI, with evidence of safety in this replacement therapy, as there was no detection of increased serum estradiol levels or hormonal levels of FSH and LH in treated patients [8]. However, the benefit is also limited to the local application area. Furthermore, the methodology used for analyzing serum estradiol levels was not homogeneous among the studies, limiting the sensitivity of the methods for detecting low concentrations of estradiol. Currently, it is known that the measurement of estradiol levels in these patients should ideally be performed using mass spectrometry. This technique is highly sensitive and provides more accurate results as there is no interference or interaction of other molecules in the assay, such as biotin, leading to more precise results. Therefore, MS is the preferred technique for evaluating breast cancer patients undergoing ovarian suppression combined with AI [9].

Several studies evaluated the impact of hormone replacement in patients with a history of breast cancer. The HABITS study, prematurely terminated due to alarming results, showed that after a 2-year follow-up, there was a more than 2-fold increase in the risk of breast cancer recurrence in women who received hormone replacement therapy with estrogen and progestogen-based compounds [12]. The Stockholm study also evaluated hormone replacement therapy in a similar setting and was halted due to the results of the HABITS study. However, in its update, recurrence rates were not as high as those found in the HABITS study [13]. Regarding hormonal treatment in patients with breast cancer, The LIBERATE study evaluated the use of tibolone in improving vasomotor symptoms caused by hormone therapy [14]. In this study, despite symptom improvement, there was a significant increase in breast cancer recurrence in patients who used the medication.

More recently, a longitudinal study conducted in Denmark evaluated postmenopausal patients with luminal breast cancer who received or did not receive adjuvant hormone therapy and who used some form of systemic or vaginal hormone replacement. This study showed a 39% increased risk of recurrence in women using aromatase inhibitors and vaginal estrogen replacement [15]. However, the absence of significant impact on the mortality makes it unclear whether the small (or absent) systemic absorption by vaginal estrogen replacement, as demonstrated by several studies [8], could negatively impact outcomes in women on aromatase inhibitors, or if the findings merely reflect a group with higher risk of relapse versus patients on tamoxifen or no endocrine therapy.

Systemic testosterone replacement therapy may also pose risks as it is converted to estradiol by aromatase enzyme activity [6]. However, by using an AI, this risk may be mitigated. For this reason, patients were required to be on AI treatment for breast cancer to prevent the conversion of testosterone to estradiol [20]. Our study demonstrated the safety of topical testosterone gel replacement, with undetectable E2 levels measured throughout the 3-month treatment protocol. Furthermore, the data showed a significant improvement in sexual satisfaction symptoms continuously throughout the treatment, with statistical significance observed between the second and third month of replacement. This finding was supported by an increase in the total score of FSFI, with statistically significant benefits observed in almost all domains assessed by the questionnaire, which are not restricted to local effects related to vaginal atrophy.

Among the evaluated patients, there was a small percentage of treatment discontinuation, mainly due to logistical difficulties and the need for outpatient evaluation to complete the questionnaire and collect the testosterone. This percentage was influenced by the fact that the majority of the study took place during the COVID-19 pandemic, which caused anxiety among patients about going to the hospital, in addition to the restrictions imposed by the isolation of infected individuals and their contacts.

Furthermore, one patient experienced a local recurrence of breast tumor in the last month of treatment, requiring surgical intervention followed by systemic treatment. This patient had E2 measurements consistently below 2.7 pg/mL throughout the treatment and was already under observation for a suspicious lesion months before entering the protocol, indicating that her recurrence was not associated with the use of the treatment.

Despite the positive results regarding the safety of testosterone replacement and the significant improvement observed in the total scores and key domains evaluated by the FSFI during the treatment, our study has several limitations. Firstly, the number of treated patients is small due to the pilot nature of the study, aimed at determining the relative safety regarding serum estradiol levels. Secondly, the follow-up period is short to assess possible recurrences and long-term benefits since the patients were treated for only three months per the protocol. Thirdly, the absence of a placebo group precludes a formal analysis of the potential benefits of the medication. However, the data obtained justify the conduction of a larger study with participant blinding and the inclusion of a control group treated with placebo gel. Fourthly, our study did not measure serum testosterone for pharmacokinetic and pharmacodynamic evaluation due to logistical and cost limitations. Lastly, the study focused on the patients’ sexual function quality and did not include validated questionnaires for other domains of quality of life that may be related to hormonal suppression. Nevertheless, the primary outcome of the study was achieved, and promising results were obtained regarding symptom improvement.

The results obtained in the study justify the implementation of larger-scale protocols to assess the role of this treatment in improving the quality of life of breast cancer patients and its safety regarding hormonal levels and potential long-term breast cancer recurrence.

## Conclusions

Our data demonstrate the maintenance of undetectable serum levels of estradiol during the 3-month use of topical testosterone. The study also suggests efficacy in improving sexual symptoms such as libido and vaginal dryness in patients with breast cancer undergoing ovarian suppression with aromatase inhibitors.

## Data Availability

No datasets were generated or analysed during the current study.

## Abbreviations

E2: Estradiol by Mass Spectrometry
MS: Mass Spectrometry
FSFI: Female Sexual Function Index
AI: Aromatase Inhibitor
DFS: Disease-Free Survival
ICF: Informed Consent Form
